# Metabolic profiles in community-acquired pneumonia: developing assessment tools for disease severity

**DOI:** 10.1186/s13054-018-2049-2

**Published:** 2018-05-14

**Authors:** Pu Ning, Yali Zheng, Qiongzhen Luo, Xiaohui Liu, Yu Kang, Yan Zhang, Rongbao Zhang, Yu Xu, Donghong Yang, Wen Xi, Keqiang Wang, Yusheng Chen, Shuchang An, Zhancheng Gao

**Affiliations:** 1 Department of Respiratory and Critical Care Medicine, Peking University People’s Hospital, Beijing, 100044 China; 2 National Protein Science Technology Center, Tsinghua University, Beijing, China; 3 School of Life Sciences, Tsinghua University, Beijing, China; 4 CAS Key Laboratory of Genome Sciences and Information, Beijing Institute of Genomics, Chinese Academy of Sciences, Beijing, China; 5 National Engineering Research Center for Beijing Biochip Technology, Beijing, China; 6 Department of Respiratory Medicine, Fujian Provincial Hospital, Fuzhou, China; 7 Department of Respiratory Medicine, First Hospital of Tsinghua University, Beijing, China

**Keywords:** Community-acquired pneumonia, Metabolomics, Severity, Biomarker, Liquid chromatography–mass spectrometry

## Abstract

**Background:**

This study aimed to determine whether community-acquired pneumonia (CAP) had a metabolic profile and whether this profile can be used for disease severity assessment.

**Methods:**

A total of 175 individuals including 119 CAP patients and 56 controls were enrolled and divided into two cohorts. Serum samples from a discovery cohort (*n* = 102, including 38 non-severe CAP, 30 severe CAP, and 34 age and sex-matched controls) were determined by untargeted ultra-high-performance liquid chromatography with tandem mass spectrometry (LC-MS/MS)-based metabolomics. Selected differential metabolites between CAP patients versus controls, and between the severe CAP group versus non-severe CAP group, were confirmed by targeted mass spectrometry assays in a validation cohort (*n* = 73, including 32 non-severe CAP, 19 severe CAP and 22 controls). Pearson’s correlation analysis was performed to assess relationships between the identified metabolites and clinical severity of CAP. The area under the curve (AUC), sensitivity and specificity of the metabolites for predicting the severity of CAP were also investigated.

**Results:**

The metabolic signature was markedly different between CAP patients and controls. Fifteen metabolites were found to be significantly dysregulated in CAP patients, which were mainly mapped to the metabolic pathways of sphingolipid, arginine, pyruvate and inositol phosphate. The alternation trends of five metabolites among the three groups including sphinganine, p-Cresol sulfate, dehydroepiandrosterone sulfate (DHEA-S), lactate and l-arginine in the validation cohort were consistent with those in the discovery cohort. Significantly lower concentrations of sphinganine, p-Cresol sulfate and DHEA-S were observed in CAP patients than in controls (*p* < 0.05). Serum lactate and sphinganine levels were positively correlated with confusion, urea level, respiratory rate, blood pressure, and age > 65 years (CURB-65), pneumonia severity index (PSI) and Acute Physiology and Chronic Health Evaluation II (APACHE II) scores, while DHEA-S inversely correlated with the three scoring systems. Combining lactate, sphinganine and DHEA-S as a metabolite panel for discriminating severe CAP from non-severe CAP exhibited a better AUC of 0.911 (95% confidence interval 0.825–0.998) than CURB-65, PSI and APACHE II scores.

**Conclusions:**

This study demonstrates that serum metabolomics approaches based on the LC-MS/MS platform can be applied as a tool to reveal metabolic changes during CAP and establish a metabolite signature related to disease severity.

**Trial registration:**

ClinicalTrials.gov, NCT03093220. Registered retrospectively on 28 March 2017.

**Electronic supplementary material:**

The online version of this article (10.1186/s13054-018-2049-2) contains supplementary material, which is available to authorized users.

## Background

Community-acquired pneumonia (CAP) is a major cause of infection-associated death worldwide, with an incidence of 30–50% in adults [1]. Due to the complexity and heterogeneity of the disease, diagnosis of CAP, especially for severe CAP, remains a clinical challenge [2]. Failure to provide timely treatment may result in water, electrolyte and acid–base balance disorders, causing multiple organ dysfunction and even septic shock in critically ill patients [3, 4]. Therefore, a timely diagnosis, assessment of the severity of CAP and initiation of appropriate treatment can improve patients’ outcomes.

Biomarkers can facilitate early severity assessment of diseases, as well as help predict treatment response and develop new insights into ongoing pathophysiological processes [2, 5]. In recent years, the focus of the discovery of CAP biomarkers has been increasingly directed towards molecular expression profiles, including gene and protein expression biomarkers in body fluids, for the diagnosis and clinical management of pneumonia [2, 6–10]. Further downstream in the biologic system, however, are small metabolites such as amino acids, carbohydrates or lipids, some of which play important roles in homeostasis and disease states, contributing to processes such as redox balance, oxidative stress, signalling, apoptosis and inflammation; these metabolites can therefore provide a more relevant and amplified signature in CAP [11]. A few metabolomics studies have offered a powerful approach for biomarker discovery and for elucidating underlying mechanisms of pneumonia [12–14], but none of the studies so far has been focused on changes in the metabolic profiles in CAP patients with different severity, and none had gone beyond the discovery phase.

In the current study, untargeted metabolomics research using liquid chromatography with tandem mass spectrometry (LC-MS/MS) was performed to identify CAP-related metabolic signatures. The identified metabolites were evaluated in the validation cohort by targeted assays. The relationship between the identified metabolites and the clinical severity of CAP as well as the determining performance for severe CAP were then investigated.

## Methods

### Study design

The study samples were obtained from the multi-centre CAP biological specimen bank of the respiratory and critical care medicine department of Peking University People’s Hospital (PKUPH). All of the obtained CAP samples were originally collected from patients hospitalized in the respiratory medicine department or intensive care unit (ICU) of six hospitals in China from January 2013 to February 2017. The study was approved by the Institutional Review Board of the PKUPH (No. 2011-83) and registered at ClinicalTrials.gov. Written informed consent was obtained from each participant.

The diagnosis criteria for CAP included the following [15]: symptom onset began in communities; presenting with clinical manifestations of pneumonia (recent appearance of cough, expectoration or exacerbated symptoms of the previous respiratory diseases, accompanied by or without chest pain, dyspnoea or haemoptysis; fever; signs of pulmonary consolidation and/or moist rales in auscultation; and peripheral white blood cell (WBC) count > 10 × 10^9^/L or < 4 × 10^9^/L); and a new pulmonary infiltrate on chest radiograph.

Severe CAP was diagnosed by the presence of at least one major criterion or at least three minor criteria outlined by the American Thoracic Society [16]. The main criteria included: requirement for invasive mechanical ventilation; and occurrence of septic shock with the need for vasopressors. The minor criteria were as follows: respiratory rate ≥ 30 breaths/min; PaO_2_/FiO_2_ ratio ≤ 250; multi-lobar infiltrates; confusion/disorientation; uraemia (BUN level ≥ 20 mg/dl); leucopenia (WBC < 4 × 10^9^/L) as a result of infection; thrombocytopenia (platelet count < 100 × 10^9^/L); hypothermia (core temperature < 36 °C); and hypotension requiring aggressive fluid resuscitation.

Patients with evidence of nosocomial infection, active pulmonary tuberculosis, malignancy, severe immunosuppression, non-infectious interstitial lung disease, pulmonary embolism and pregnancy were excluded. During the same period, healthy volunteers or subjects who visited the outpatient department of PKUPH for a routine health examination and were without CAP were also enrolled as a control group. Baseline clinical parameters of CAP patients were obtained from their clinical records and then uploaded to an online case management system of Severe Acute Respiratory Infectious Disease in China by their physicians. The primary endpoint representative of severe CAP was defined as death within 30 days following inclusion. The secondary endpoint was invasive mechanical ventilation or ICU admission within 30 days following study inclusion. Outcomes were assessed at hospital discharge, and by structured telephone interviews at 30 days following inclusion.

### Sample collection and preparation

All of the participants were in an overnight fasting state, and 5 ml of peripheral venous blood was taken in the morning within 48 h of hospital admission. The blood was then allowed to clot for 30 min at 4 °C, followed by centrifugation at 1500 × *g* for 15 min. The serum supernatant was then collected and stored at − 80 °C until further use.

Before testing, the serum samples were thawed at 4 °C on ice. Then 400 μl of pre-chilled methanol (containing l-tryptophan-d5, l-glutamine-^13^C5, l-glutamine-^15^N2, terfenadine and propranolol as internal standards) was added to 100 μl aliquots of the serum samples to precipitate the proteins. After vortex-blending for 15 s and incubation at − 80 °C for 1 h, the mixture was centrifuged at 13,400 × *g* for 20 min at 4 °C. The supernatant was then transferred to a new 1.5-ml Eppendorf tube and dried before storage in the − 80 °C freezer. A pooled quality control (QC) sample solution was prepared by combining equal volumes of serum from each sample and treated using the same procedure already described. Before experimental sample analyses, six QCs were injected to stabilize the instrument. The analysis sequence of the samples to be tested was randomized with a QC sample between every 10 experimental samples. QC samples were used to monitor the reliability of the whole experiment including sample preparation and LC-MS/MS sequence runs.

### Untargeted LC-MS/MS analysis and metabolite identification

Details of liquid chromatography–mass spectrometry-based metabolomics analyses are described in Additional file 1. Samples were analysed using an Ultimate 3000 UHPLC (Dionex) system coupled to a Thermo Q-Exactive (Orbitrap) mass spectrometer (Thermo Fisher Scientific, San Jose, CA, USA).

Two levels of identification were performed simultaneously using TraceFinder (Thermo Fisher Scientific, San Jose, CA, USA). Metabolites were first potentially identified according to the endogenous MS database by accurate masses. At the same time, the metabolites that matched with the spectra in the fragment database were confirmed at the tandem mass spectrometry level. For precursor and fragment matching, 10 ppm and 15 ppm mass tolerance was applied. Moreover, a 0.25-min retention time shift was allowed for quantification.

### Multivariate data analysis

Multivariate statistical analysis for the MS/MS data was performed using SIMCA 14.0 software (Umetrics AB, Umea, Sweden). Unsupervised principal component analysis (PCA) was employed to assess the quality, homogeneity, outlier identification and dominating trends of the group separation inherent in the dataset. A supervised orthogonal partial least squares discriminant analysis (OPLS-DA) was applied to distinguish between the classes and to identify the differentially expressed variables. Corresponding variable importance in the projection (VIP) values and S-plot were generated in the OPLS-DA model. The quality of the multivariate statistical analysis (MVA) models was evaluated by cross-validation analysis of variance (CV-ANOVA), *R*^2^*Y* and *Q*^2^ values. Student’s *t* test was used to determine the significance of each metabolite between two groups, and the relevant false discovery rates (FDR) based on the *p* values were estimated. Variables with covariance > 0.1 on the S-plot, VIP > 1 and FDR < 0.05 were considered the highest potential metabolites that could discriminate CAP from controls and for severity assessment. These metabolites were then identified through matching accurate mass and MS/MS spectra using in-house metabolite MS/MS databases. Pathway analysis of selected differential metabolites, a heatmap with a Euclidean distance measure of relative intensity of metabolites (logarithmic scale) and a Pearson’s correlation heatmap were generated using MetaboAnalyst 3.0 (http://www.metaboanalyst.ca/; Wishart Research Group, University of Alberta, USA) [17].

### Validation and quantification of metabolites

Targeted metabolomics and absolute quantification was performed for differential metabolites identified between CAP patients and controls under the same experimental conditions and procedures in the validation cohort. Concentrations (μg/ml) of the metabolites were calculated by area response ratio (analyte peak area/isotopically labelled internal standard peak area) from independent calibration curves for each metabolite.

### Statistical analysis

Values for categorical variables were described as percentages and those for continuous variables were expressed as median (interquartile range (IQR)). Comparisons between groups were performed using the Mann–Whitney *U* test or Kruskal–Wallis test for continuous variables, and the chi-square test or Fisher’s exact test for categorical variables, as appropriate. Pearson’s correlation coefficients (*r*) were calculated to assess the strength and direction of linear relationships between the identified metabolites, and clinical parameters included WBC, percentage of neutrophils (NE%), erythrocyte sedimentation rate (ESR), C-reactive protein (CRP), procalcitonin (PCT), confusion, urea level, respiratory rate, blood pressure, and age > 65 years (CURB-65), pneumonia severity index (PSI) and Acute Physiology and Chronic Health Evaluation II (APACHE II) scores. Goodness of the diagnostic method was evaluated using a receiver operating characteristic (ROC) curve based on multivariate logistic regression data. Data were analysed using SPSS Statistics v19.0 (IBM, NY, USA) and MedCalc software version 15.8 (MedCalc Software, Ostend, Belgium). All statistics were two-tailed and the significance level was defined at *p* < 0.05.

## Results

### Demographic and clinical characteristics of participants

**Fig. 1:**
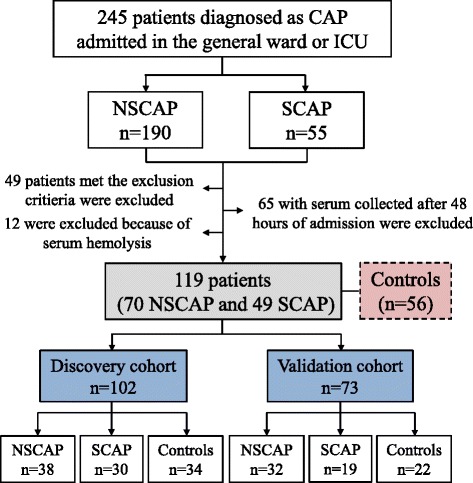
Flowchart of study population enrolment. CAP community-acquired pneumonia, ICU intensive care unit, NSCAP non-severe CAP, SCAP severe CAP

From January 2013 to February 2017, 245 patients diagnosed with CAP (190 with non-severe CAP and 55 with severe CAP) were screened. The flowchart of the study population enrolment is shown in Fig. 1. A total of 175 subjects (119 CAP patients and 56 controls) were included finally and divided into a discovery cohort (*n* = 102, including 38 non-severe CAP patients, 30 severe CAP patients, and 34 age and sex-matched controls) and a validation cohort (*n* = 73, including 32 non-severe CAP patients, 19 severe CAP patients and 22 controls). Demographic and clinical characteristics of the participants are presented in Table 1.

**Table 1:** Demographic and clinical characteristics of the 175 subjects enrolled in this study

Characteristic	Discovery cohort (*n* = 102)				Validation cohort (*n* = 73)			
	NSCAP (*n* = 38)	SCAP (*n* = 30)	Controls (*n* = 34)	*p* value	NSCAP (*n* = 32)	SCAP (*n* = 19)	Controls (*n* = 22)	*p* value
Age (years)	56.5 (45.8–64.5)	58 (43–82.3)	57 (46.3–62.5)	0.647	28 (23.3–43)	64 (54–74)	40.5 (30.8–49.3)	< 0.001
Sex, male	23 (60.5)	23 (76.7)	21 (61.8)	0.319	16 (50.0)	15 (78.9)	12 (54.5)	0.112
Smoking history	7 (18.4)	2 (6.7)	8 (23.5)	0.209	2 (6.3)	3 (15.8)	4 (18.2)	0.354
Underlying diseases								
Respiratory diseases	1 (2.6)	0 (0)	1 (2.9)	1.000	0 (0)	4 (21.1)	0 (0)	0.004
Cardiovascular diseases	3 (7.9)	6 (20.0)	9 (26.5)	0.122	0 (0)	5 (26.3)	1 (4.5)	0.002
Liver diseases	1 (2.6)	2 (6.7)	1 (2.9)	0.681	0 (0)	0 (0)	0 (0)	NA
Neurological diseases	3 (7.9)	4 (13.3)	2 (5.9)	0.621	0 (0)	3 (15.8)	0 (0)	0.016
Hyperlipidaemia	1 (2.6)	1 (3.3)	4 (11.8)	0.315	0 (0)	1 (5.3)	0 (0)	0.260
Infected pathogens								
Bacterial	6 (15.8)	10 (33.3)	NA	0.090	9 (28.1)	7 (36.8)	NA	0.547
Viral	3 (7.9)	7 (23.3)	NA	0.094	2 (6.3)	3 (15.8)	NA	0.348
Atypical pathogens	6 (15.8)	2 (6.7)	NA	0.288	11 (34.4)	2 (10.5)	NA	0.096
WBC (×10^9^/L)	7.4 (5.6–10.8)	7.7 (5.1–10.6)	NA	0.946	5.6 (4.4–7.5)	10.7 (7.1–15.6)	NA	0.001
NE%	66.4 (56.0–81.3)	83.5 (78.3–90.4)	NA	< 0.001	67.1 (63.7–77.3)	84.1 (78.8–91.6)	NA	0.002
ESR (mm/h)	47.0 (41.5–51.5)	60.0 (43.0–66.3)	NA	0.082	27.0 (17.5–45.0)	65.0 (35.0–79.0)	NA	0.009
CRP (mg/l)	51.5 (49.0–121.8)	123.0 (83.4–141.0)	NA	0.013	55.3 (22.3–82.0)	111.0 (60.6–196.5)	NA	0.008
PCT (μg/L)	0.2 (0.1–1.3)	0.5 (0.2–3.7)	NA	0.086	0.1 (0.1–0.2)	0.9 (0.4–3.1)	NA	0.009
PaO_2_/FiO_2_	379.5 (306.3–403.5)	197.5 (172.8–252.3)	NA	< 0.001	378.0 (358.0–390.8)	187.0 (153.0–219.0)	NA	< 0.001
CURB-65 score	0 (0-1)	1 (0-2)	NA	< 0.001	0 (0-0)	1 (1–2)	NA	< 0.001
PSI score	59.5 (46.8–76.0)	86.5 (59.3–120.8)	NA	0.001	35.5 (25.0–54.5)	87.0 (60.0–118.0)	NA	< 0.001
APACHE II score	4 (3-6)	11.5 (7-16)	NA	< 0.001	3 (2-5)	10 (6–15)	NA	< 0.001
Drug treatment								
Antibiotics	37 (97.4)	30 (100)	NA	1.000	32 (100)	19 (100)	NA	NA
Antiviral drugs	2 (5.3)	12 (40.0)	NA	0.001	2 (6.3)	4 (21.1)	NA	0.179
Corticosteroids	2 (5.3)	7 (23.3)	NA	0.037	1 (3.1)	4 (21.1)	NA	0.058
Vasopressors	0 (0)	8 (26.7)	NA	0.001	0 (0)	5 (26.3)	NA	0.005
Non-invasive ventilation	0 (0)	16 (53.3)	NA	< 0.001	2 (6.3)	9 (47.4)	NA	< 0.001
Invasive ventilation	0 (0)	11 (36.7)	NA	< 0.001	0 (0)	5 (26.3)	NA	< 0.001
ECMO	0 (0)	1 (3.3)	NA	0.441	0 (0)	0 (0)	NA	NA
Sepsis	0 (0)	11 (36.7)	NA	< 0.001	0 (0)	9 (47.4)	NA	< 0.001
Hospital LOS (days)	11.0 (7.8–14.0)	17.5 (12.8–22.0)	NA	< 0.001	11.5 (8.0–13.8)	15.0 (11.0–24.0)	NA	0.005
ICU admission	0 (0)	15 (50)	NA	< 0.001	0 (0)	6 (31.6)	NA	0.002
In-hospital mortality	0 (0)	7 (23.3)	NA	0.002	0 (0)	3 (15.8)	NA	< 0.001
30-day mortality	0 (0)	7 (23.3)	NA	0.002	0 (0)	2 (10.5)	NA	< 0.001

Data presented as number (percentage) or median (interquartile range)

*CAP* community-acquired pneumonia, *NSCAP* non-severe CAP, *SCAP* severe CAP, *WBC* white blood cell, *NE%* percentage of neutrophils, *ESR* erythrocyte sedimentation rate, *CRP* C-reactive protein, *PCT* procalcitonin, *PaO*_*2*_*/FiO*_*2*_ ratio of arterial oxygen tension to inspired oxygen fraction, *CURB-65* confusion, urea level, respiratory rate, blood pressure, and age > 65 years, *PSI* pneumonia severity index, *APACHE II* Acute Physiology and Chronic Health Evaluation II, *ECMO* extracorporeal membrane oxygenation, *LOS* length of stay, *ICU* intensive care unit, *NA* not applicable

In the discovery cohort, there were no significant differences in terms of age, gender, underlying disease, smoking history or infected pathogens among the three groups (*p* > 0.05). NE% and the levels of serum CRP were both greater in the severe CAP group than in the non-severe group (*p* < 0.05). CURB-65, PSI and APACHE II scores in the severe CAP group were all significantly higher than in the non-severe CAP group (all *p* < 0.05). Patients with severe CAP were more frequently receiving antiviral drugs, corticosteroids, vasopressors treatment and ventilation (*p* < 0.05) during hospitalization, and also were more likely to develop sepsis and be admitted to the ICU (*p* < 0.001 for both comparisons).

In the validation cohort, patients with severe CAP were older than those with non-severe CAP (*p* < 0.001). The proportion of underling diseases such as respiratory diseases, cardiovascular diseases and neurological diseases were all significantly higher in the severe CAP group (*p* < 0.05). The in-hospital mortality and 30-day mortality in patients with severe CAP were both substantially higher compared to those with non-severe CAP (*p* < 0.001).

### Untargeted metabolomics and pathway analysis

Representative base peak chromatograms of the serum samples from subjects with non-severe CAP, subjects with severe CAP and healthy controls in the discovery cohort are shown in Additional file 2: Figure S1. A total of 820 metabolites including 367 in the election spray ionization positive (ESI+) mode and 453 in the ESI– mode were detected by untargeted LC-MS/MS analysis. Among them, 775 metabolites including 332 in the ESI+ and 443 in the ESI– mode were identified with known MS/MS information, and were subjected to PCA and OPLS-DA analysis.

**Fig. 2:**
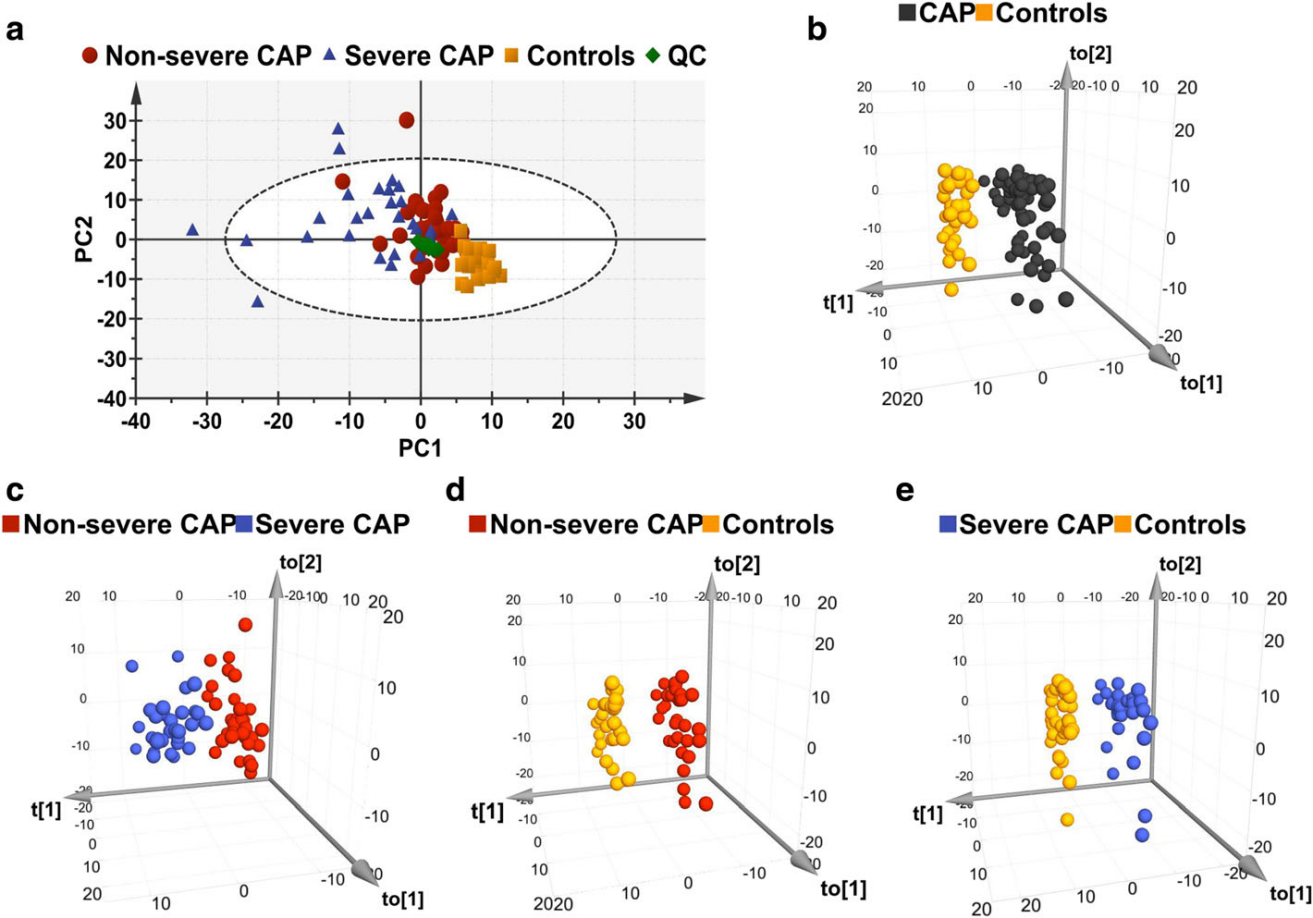
Multivariate statistical analysis of serum samples in discovery cohort. **a** PCA score plots. Five samples (four severe CAP and one non-severe CAP) are placed outside the ellipse that describes the 95% CI of Hotelling’s T-squared distribution. **b** OPLS-DA three-dimensional score plot discriminates all CAP subjects versus controls in discovery cohort (*R*^2^*Y* = 0.937, *Q*^2^ = 0.814, *p* < 0.0001). **c** OPLS-DA score plots of non-severe CAP versus severe CAP groups (*R*^2^*Y* = 0.757, *Q*^2^ = 0.465, *p* < 0.0001). **d** OPLS-DA score plots of non-severe CAP patients versus controls (*R*^2^*Y* = 0.994, *Q*^2^ = 0.955, *p* < 0.0001). **e** OPLS-DA score plots of severe CAP patients versus controls (*R*^2^*Y* = 0.996, *Q*^2^ = 0.854, *p* < 0.0001). *R*^2^*Y* represents goodness of fit, *Q*^2^ represents goodness of prediction, *p* value shows significance level of the model. CAP community-acquired pneumonia, PC principal component, QC quality control

PCA performed on all of the subjects revealed differences in the metabolic profiles of patients with non-severe CAP and severe CAP, and controls (Fig. 2a). QC samples are tightly clustered on the PCA plot, indicating the good analytical repeatability and stability of the instruments. The OPLS-DA score plot showed a clear separation between the CAP subjects and controls (Fig. 2b), with good fitting and predictive performances (*R*^2^*Y* = 0.937, *Q*^2^ = 0.814). Any other comparison of two groups between patients with non-severe CAP versus severe CAP (Fig. 2c), patients with non-severe CAP versus controls (Fig. 2d), and patients with severe CAP versus controls (Fig. 2e) showed clear discrimination.

**Fig. 3:**
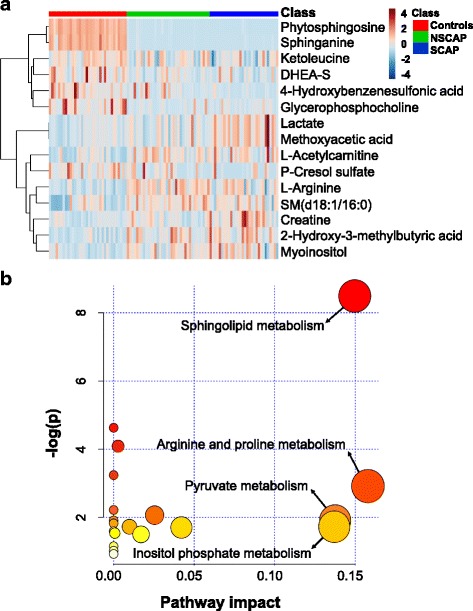
Fifteen metabolites dysregulated in CAP compared to controls. **a** Hierarchical cluster heatmap of 15 metabolites in three groups. Row represents metabolites and column represents individual samples. Green, red and blue represent non-severe CAP (NSCAP), severe CAP (SCAP) and controls, respectively. Greater brown indicates higher relative intensity of metabolites, while light blue indicates lower intensity. **b** Metabolic pathway analysis of 15 metabolites changed in CAP. Node colour based on *p* value and node radius determined based on pathway impact values. CAP community-acquired pneumonia, DHEA-S dehydroepiandrosterone sulfate

With a covariance (absolute *p*) > 0.1 on the S-plot (Additional file 2: Figure S2), VIP > 1 and FDR < 0.05 for the comparison between CAP and controls, 15 metabolites were found to be significantly dysregulated in CAP (Table 2). The coefficient of variation (CV) of the 15 metabolites varied from 3.19 to 29.79% with a median of 7.73%, which indicated the robustness of the metabolomics platform. The relative intensity of palmitoyl sphingomyelin (SM(d18:1/d16:0), creatine, l-arginine, lactate, myoinositol, 2-hydroxy-3-methylbutyric acid, methoxyacetic acid and l-acetylcarnitine was increased in CAP patients and that of phytosphingosine, sphinganine, 4-hydroxybenzenesulfonic acid, ketoleucine, glycerophosphocholine, dehydroepiandrosterone sulfate (DHEA-S) and p-Cresol sulfate was decreased. A heatmap and the box–whisker plots indicating the relative intensity of these metabolites in the three groups are displayed in Fig. 3a and Additional file 2: Figure S3, respectively. Pathway analysis of the 15 identified metabolites dysregulated in the CAP patients mainly revealed four pathways that were affected: sphingolipid metabolism, arginine and proline metabolism, pyruvate metabolism, and inositol phosphate metabolism (Fig. 3b).

**Table 2:** Fifteen metabolites discriminating CAP from controls in the discovery cohort

Metabolite/pathway	ESI mode	Mass (*m/z*)	RT (min)	VIP	FDR	FC^a^	CV%^b^
Sphingolipid metabolism							
Phytosphingosine	Pos	318.3003	3.7428	5.1690	2.99×10^–18^	0.2005	8.45
Sphinganine	Pos	302.3054	3.7117	3.5729	3.74×10^–12^	0.2276	10.90
Palmitoyl sphingomyelin (SM(d18:1/16:0))	Pos	703.5754	5.9542	4.2044	1.31×10^–5^	1.6366	29.79
Arginine and proline metabolism							
Creatine	Pos	132.0773	9.4855	2.6225	8.71×10^–4^	1.6254	9.13
l-Arginine	Pos	175.1195	11.6748	3.6558	1.18×10^–6^	1.3980	5.83
Pyruvate metabolism							
Lactate	Neg	89.0239	6.9559	3.4407	1.04×10^–3^	1.3259	5.50
Inositol phosphate metabolism							
Myoinositol	Neg	179.0561	8.2464	3.8451	6.54×10^–5^	1.4138	6.81
2-Hydroxy-3-methylbutyric acid	Neg	117.0557	4.7190	3. 6752	1.63×10^–6^	2.0376	18.88
Other metabolisms							
4-Hydroxybenzenesulfonic acid	Neg	172.9914	0.7999	4.6749	1.19×10^–4^	0.3932	3.19
Methoxyacetic acid	Neg	89.0239	6.9220	3.4407	1.04×10^–3^	1.3259	5.50
Ketoleucine	Neg	129.0552	2.4610	6.8758	2.65×10^–3^	0.8778	7.01
l-Acetylcarnitine	Pos	204.1230	8.3173	3.9173	2.91×10^–5^	2.1304	27.89
Glycerophosphocholine	Pos	258.1101	10.8398	11.0112	6.98×10^–7^	0.2979	24.21
DHEA-S	Neg	367.1579	0.9317	3.6010	7.68×10^–5^	0.5623	4.65
p-Cresol sulfate	Neg	187.0065	0.7839	9.9153	1.18×10^–2^	0.5306	3.54

*CAP* community-acquired pneumonia, *ESI* election spray ionization, *RT* retention time, *VIP* variable importance in the projection, *FDR* false discovery rate, *FC* fold change, *CV* coefficient of variation, *Pos* positive, *Neg* negative, *DHEA-S* dehydroepiandrosterone sulfate

^a^Ratio of relative high intensity present in CAP patients to controls

^b^CV% for quality control sample

Eight metabolites could distinguish patients with severe CAP from those with non-severe CAP and are presented in Additional file 3: Tables S1 and S4. The levels of creatine, lactate and methoxyacetic acid gradually increased with CAP severity, while those of DHEA-S and 4-hydroxybenzenesulfonic acid showed the opposite pattern. In addition, lower relative abundances of phytosphingosine and sphinganine were observed in the non-severe CAP group compared to the severe CAP group and controls. The metabolites changes between severe CAP patients versus controls, and between non-severe CAP patients versus controls, and are summarized in Additional file 3: Tables S2–S4.

### Targeted metabolomics and absolute quantification

**Fig. 4:**
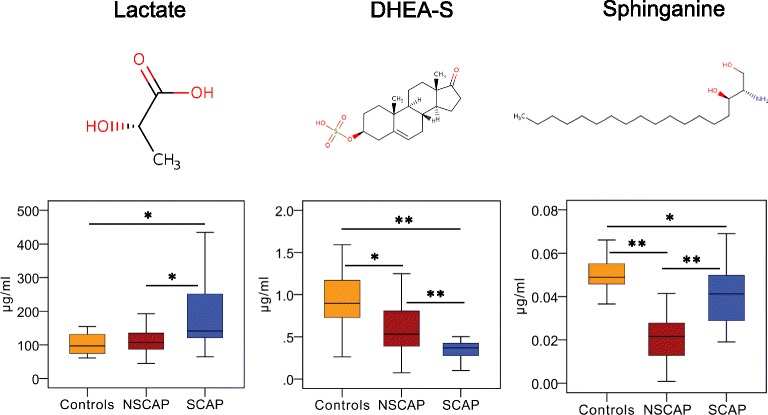
Three metabolites identified that can discriminate severe CAP from non-severe CAP in validation cohort. Chemical structures of three metabolites. Box–whisker plots of concentrations of three metabolites in three groups. Horizontal line represents median; bottom and the top of box represent 25th and the 75th percentiles; whiskers represent 5% and 95% percentiles. *p < 0.01, **p < 0.001. CAP community-acquired pneumonia, DHEA-S dehydroepiandrosterone sulfate, NSCAP non-severe CAP, SCAP severe CAP

Targeted metabolomics were performed in another population to validate the observed trend of the identified 15 differential metabolites between CAP patients and controls from the discovery cohort. The results showed that the secondary mass spectra of methoxyacetic acid were representative of its isomer, lactate. Six metabolites including phytosphingosine, palmitoyl sphingomyelin (SM(d18:1/16:0)), myoinositol, 2-hydroxy-3-methylbutyric acid, 4-hydroxybenzenesulfonic acid and ketoleucine exhibited low responses in the targeted assays, so only eight metabolites including sphinganine, lactate, DHEA-S, l-arginine, p-Cresol sulfate, l-acetylcarnitine, creatine and glycerophosphocholine were ultimately validated and absolute quantified. The alternation trends of five metabolites among three groups including sphinganine, p-Cresol sulfate, DHEA-S, lactate and l-arginine in the validation cohort were consistent with those in the discovery cohort. Compared with controls, CAP patients showed lower concentrations of the first three metabolites (*p* < 0.001) and higher concentrations of the last two metabolites (*p* < 0.05) (Additional file 3: Table S5). Lactate levels were found to increase with CAP severity, while DHEA-S levels reduced gradually with increasing severity of CAP (Fig. 4 and Table 3).

**Table 3:** Eight metabolite concentrations in severe CAP and non-severe CAP patients of the validation cohort

Metabolite	Concentration (μg/ml)		*p* value	Tendency
	SCAP (*n* = 19)	NSCAP (*n* = 32)		
Sphinganine	0.041 (0.028–0.050)	0.022 (0.012–0.028)	< 0.001	Controls > SCAP > NSCAP
Creatine	5.420 (3.447–8.711)	2.481 (1.739–3.767)	< 0.001	SCAP > controls > NSCAP
l-Arginine	5.043 (4.105–5.753)	5.905 (4.622–7.047)	0.016	NSCAP > SCAP > controls
Lactate	141.343 (120.670–273.794)	107.224 (86.814–137.551)	0.001	SCAP > NSCAP > controls
l-Acetylcarnitine	0.076 (0.029–0.225)	0.063 (0.042–0.098)	0.667	Controls > SCAP > NSCAP
Glycerophosphocholine	4.438 (2.596–8.170)	5.732 (3.048–7.212)	0.181	Controls > NSCAP > SCAP
DHEA-S	0.370 (0.229–0.425)	0.532 (0.379–0.812)	< 0.001	Controls > NSCAP > SCAP
p-Cresol sulfate	0.109 (0.004–0.801)	0.073 (0.012–0.203)	< 0.001	Controls > SCAP > NSCAP

Data presented as median (interquartile range)

*CAP* community-acquired pneumonia, *SCAP* severe CAP, *NSCAP* non-severe CAP, *DHEA-S* dehydroepiandrosterone sulfate

### Relationship to clinical parameters and assessment performance

**Fig. 5:**
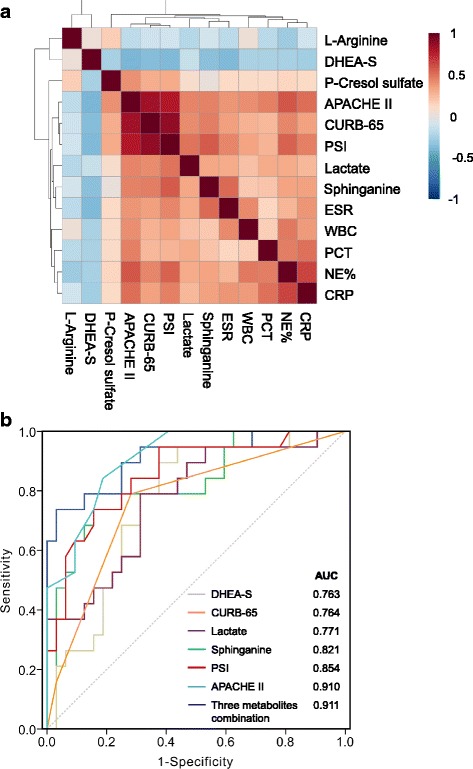
Correlations of five metabolites with clinical parameters and assessment performance. **a** Pearson’s correlation heatmap of five serum metabolites and clinical parameters. Greater intensities of brown and blue indicate higher positive or negative correlations, respectively. Resulting correlation matrix presented in Additional file 3: Table S6. **b** ROC curve analysis of various parameters for discrimination of severe CAP from non-severe CAP. DHEA-S dehydroepiandrosterone sulfate, APACHE II Acute Physiology and Chronic Health Evaluation II, CURB-65 confusion, urea level, respiratory rate, blood pressure, and > 65 years, PSI pneumonia severity index, ESR erythrocyte sedimentation rate, WBC white blood cell, PCT procalcitonin, NE*%* percentage of neutrophils, CRP C-reactive protein, AUC area under the curve

The relationships between five metabolites—sphinganine, p-Cresol sulfate, DHEA-S, lactate and l-arginine—and the clinical parameters were investigated by Pearson’s correlation analysis (Fig. 5a). The resulting correlation matrix is presented in Additional file 3: Table S6. Sphinganine was found to be positively correlated with CURB-65 (*r* = 0.456, *p* = 0.001), PSI (*r* = 0.570, *p* < 0.001) and APACHE II (*r* = 0.442, *p* = 0.001) scores. It was also positively related with NE% (*r* = 0.336, *p* = 0.016), ESR (*r* = 0.519, *p* < 0.001) and CRP (*r* = 0.340, *p* = 0.015). Lactate and p-Cresol sulfate were observed to show a positive correlation with CURB-65, PSI and APACHE II (all *p* < 0.05) scores, whereas DHEA-S was negatively related with the three scoring systems (all *p* < 0.05). l-Arginine was not correlated with these clinical parameters except for NE% (*r* = − 0.283, *p* = 0.044).

ROC analysis was performed to investigate whether the five identified metabolites could be efficiently utilized for building a sensitive biosignature of severe status in CAP. As shown in Table 4 and Fig. 5b, sphinganine (AUC 0.821, *p* < 0.001) exhibited a better performance than CURB-65 score (AUC 0.764) to predict the severity of CAP, with a sensitivity of 73.7% and specificity of 84.4%. The AUCs for lactate and DHEA-S were 0.771 and 0.763, respectively. The three metabolites were not superior to PSI (AUC 0.854) and APACHE II (AUC 0.910) scores, while the multiple logistic regression analysis revealed that a combination of them had an AUC value of 0.911 (95% CI 0.825–0.998), with 83.7% sensitivity and 96.9% specificity, indicating that they can serve as a metabolite panel of potential biomarkers for assessing CAP severity. The optimal cut-off value of sphinganine, DHEA-S and lactate to discriminate severe CAP from non-severe CAP was calculated to be 0.029 μg/ml, 0.431 μg/ml and 120.021 μg/ml, respectively. Regarding the remaining two metabolites, l-arginine showed a poor ability in predicting severe CAP (AUC = 0.689, *p* = 0.025) and p-Cresol sulfate had no prognostic value for CAP severity prediction (AUC = 0.503, *p* = 0.969).

**Table 4:** Areas under the curve of variable parameters for determining the severity of CAP

Parameter	Cut-off value	AUC	Sensitivity	Specificity	*p* value	95% CI	
						Lower limit	Higher limit
Sphinganine (μg/ml)	> 0.029	0.821	0.737	0.844	< 0.001	0.698	0.943
DHEA-S (μg/ml)	< 0.431	0.763	0.895	0.625	0.002	0.631	0.896
Lactate (μg/ml)	> 120.021	0.771	0.789	0.687	0.001	0.637	0.905
Three metabolite combination	–	0.911	0.837	0.969	< 0.001	0.825	0.998
ESR	–	0.748	0.706	0.905	0.009	0.565	0.931
CRP	–	0.749	0.706	0.870	0.004	0.582	0.917
PCT	–	0.832	0.875	0.875	< 0.001	0.654	1.000
CURB-65	–	0.764	0.789	0.719	0.002	0.625	0.903
PSI	–	0.854	0.737	0.844	< 0.001	0.728	0.937
APACHE II	–	0.910	0.842	0.813	< 0.001	0.796	0.972

*CAP* community-acquired pneumonia, *AUC* area under the curve, *CI* confidence interval, *DHEA-S* dehydroepiandrosterone sulfate, *ESR* erythrocyte sedimentation rate, *CRP* C-reactive protein, *PCT* procalcitonin, *CURB-65* confusion, urea level, respiratory rate, blood pressure, and age > 65 years, *PSI* pneumonia severity index, *APACHE II* Acute Physiology and Chronic Health Evaluation II

## Discussion

The current study describes a novel application of metabolomics in determining the metabolic profile and severity assessment of CAP. Untargeted metabolic analysis could clearly discriminate CAP patients from the age and sex-matched controls, suggesting that CAP causes a significant disruption in biochemical homeostasis. Fifteen metabolites showed key differences between CAP patients and controls in our discovery cohort. Pathway analysis revealed that these dysregulated metabolites were potentially related to the metabolic pathways of sphingolipid, arginine and proline, pyruvate and inositol phosphate. After analysing the relationships between the identified metabolites with clinical parameters, and calculating the diagnostic efficacy of these metabolites using ROC, we concluded that three metabolites—sphinganine, lactate and DHEA-S, related to the severity of CAP—might represent a panel of potential small molecule biomarkers for assessing CAP severity.

Some of the preceding metabolomics studies aiming to identify biomarkers associated with diseases adopted the criteria based on fold change (FC), VIP, FDR or *p* value to select the differential metabolites [18, 19]. The S-plot generated from OPLS-DA analysis visualizes both the covariance and correlation between the metabolites and the modelled class designation, and thereby it can help in identifying statistically significant and potentially biochemically significant metabolites, based both on contributions to the model and their reliability [20]. Therefore, we strictly limited the criteria to select the differential metabolites in this study, based on the combination of VIP > 1, FDR < 0.05 and covariance > 0.1 on the S-plot, and finally identified 15 differential metabolites between CAP patients and the control group.

Among the identified potential metabolic biomarkers for CAP, the levels of sphinganine in the serum of CAP patients is lower than for controls, and it was observed positively correlated with NE%, ESR and CRP, so it might reflect the presence of an infection and inflammatory response. The serum concentration of sphinganine in severe CAP patients is higher than that in non-severe CAP patients, and lower than controls, but the AUC to distinguish between non-severe CAP and severe CAP was 0.821 and it correlated positively with CURB-65, PSI and APACHE II scores. We therefore considered that it could roughly be related to the severity of CAP. Sphinganine is a major component of sphingolipids, which are one of the active constituents of the mucous secreted by the alveolar epithelium, which protects the lung tissue from invading pathogens [21].One of the important aspects of sphingolipids and their primary and intermediate metabolites is their interconvertible nature, which enables them to both integrate and regulate a plethora of cellular functions [22, 23]. Previous studies have shown that *Mycoplasma pneumonia* infection in the lungs results in the induction of autoantibody production against glycosphingolipids, suggesting the involvement of sphingolipids in promoting lung inflammation [24, 25]. Moreover, compelling evidence indicates that certain pulmonary pathogens such as *Chlamydia* cause the trafficking of sphingolipids from the trans-Golgi apparatus towards the inclusion membrane to ensure their intracellular survival, which contributes to the immune-evading mechanisms of bacteria [26]. Therefore, we speculated that sphingolipids might be involved in pulmonary inflammation during infection.

We found that the DHEA-S concentration was lower in the CAP patients than in controls, and its levels were inversely correlated with disease severity. Dehydroepiandrosterone (DHEA) is the most abundant adrenal steroid hormone in humans, and DHEA-S, the sulphated ester of DHEA, is the hydrophilic storage form bound to albumin in the bloodstream [27]. It has been shown that DHEA modulates the function of the immune system [28]. A previous study reported that, upon activation by a variety of stimuli such as mitogens or antigens, CD4^+^ T cells in healthy adults pre-treated with DHEA produced significantly greater amounts of interleukin (IL)-2 and mediated more potent cytotoxicity than CD4^+^ T cells not pre-treated with DHEA [29]. In another study, patients with tuberculosis showed decreased DHEA levels compared with healthy control subjects, and patients with the lowest DHEA activity showed the highest disease severity [30]. Moreover, biological activity for DHEA-S was found to be able to enhance the activity of human neutrophils. Therefore, diminished levels of DHEA-S could have adverse effects, especially in relation to susceptibility to bacterial infection [31]. Based on these findings, we speculated that suppression of DHEA-S levels in patients with severe CAP might further downregulate the immune response to foreign pathogens. This indicates that DHEA or DHEA-S shows potential for use in alternative therapies, as a supplement to currently used long-term antibiotic treatment regimens, or as a preventive strategy against disease recurrence.

During lung infection, glycolysis results in the production of pyruvate, which is converted by lactate dehydrogenase to lactate under anaerobic conditions [32]. Serum lactate levels have been use for many years in the assessment of tissue hypoxia and perfusion status, and are often used clinically as an indicator of the severity of sepsis and of patient outcomes in sepsis/septic shock [33–35]. A previous study showed that a lactate level in arterial blood gas of > 1.8 mmol/L at admission could predict a need for mechanical ventilation, vasopressors, ICU admission or hospital mortality in patients with CAP [36]. In our study, serum lactate levels were slightly increased in the patients with non-severe CAP and highly increased in the patients with severe CAP. The increase might reflect the state of anaerobic glycolysis of glucose during severe pulmonary infections. Furthermore, ROC analysis suggested that high serum lactate levels could predict the occurrence of severe CAP, with an optimal cut-off of > 120.021 μg/ml (1.34 mmol/L). Lactate could therefore be considered as a potential metabolic biomarker for the assessment of risk of severe CAP.

It is important to note that the predictability for separation of severe CAP from the controls (*Q*^2^ = 0.854) in the OPLS-DA model is not better than the separation for non-severe CAP from controls (*Q*^2^ = 0.955), while we expect to observe more alteration in severe CAP than non-severe CAP and better separation from controls. This result possibly was related to the high heterogeneity of the metabolic status of the severe CAP group in our study, which might have an impact on the degree of distinction and the predictability for separation between severe CAP and the control group.

Our independent validation study adds tremendous validity to our initial findings; nevertheless, biomarker discovery is no small undertaking and typically requires years of validation testing before the application phase is reached [11]. This study is just an initial step in CAP severity assessment using a metabolomics approach. It has certain limitations. First of all, due to the small sample size of non-survivors in our validation cohort, the prognostic predicative value of the identified potential metabolic biomarkers for CAP could not be determined precisely. Next, the proportion of ventilation in patients with severe CAP was significantly higher than that in non-severe CAP in this study. The ventilation condition might affect the metabolomics pictures, so additional studies would be needed to evaluate the serum metabolic status of patients with CAP before mechanical ventilation. Subsequently, the convalescence-phase serum samples from the CAP patients in our study were not obtained for further analyses, and therefore we were unable to identify changes in metabolite concentrations over time, which might be predictive of disease progression, therapeutic response or clinical outcome. Additionally, we could not specifically elucidate the roles of the identified metabolites in CAP pathogenesis. Elaborate studies consisting of a larger external cohort are needed to validate the utility of the identified potential biomarkers, and research aimed at achieving the long sought-after goal—integration of multi-omics data in CAP research—will certainly support the development of precision medicine.

## Conclusions

Our results suggest that metabolomics approaches based on LC-MS/MS can be successfully used to reveal metabolic changes in CAP and establish a metabolite signature related to disease severity. The potential molecular metabolites identified in this study and their relevant roles may provide valuable clues for future research on CAP biomarker discovery and for the development of precision medicine for patients with CAP.

## Additional files

Additional file 1: Supplemental methods. Liquid chromatography–mass spectrometry (LC-MS) analysis. (DOCX 19 kb)

Additional file 2: Supplemental Figures. **Figure S1.** Metabolite base peak chromatograms of serum samples from a patient in three groups: a non-severe CAP; b severe CAP; c controls. **Figure S2.** S-plots identifying putative biomarkers on the basis of OPLS-DA models: a CAP patients versus controls; b severe CAP versus non-severe CAP patients. **Figure S3.** Box–whisker plots of relative intensity of 15 metabolites changed in CAP patients compared to controls. Horizontal line represents median; bottom and top of the box represent 25th and the 75th percentiles; whiskers represent 5% and 95% percentiles. *FDR < 0.05, **FDR < 0.001. NSCAP non-severe CAP, SCAP severe CAP. (DOCX 7213 kb)

Additional file 3: Supplemental tables. **Table S1.** Changes in eight metabolites between severe and non-severe CAP patients in discovery cohort. **Table S2.** Changes in 17 metabolites between severe CAP patients and controls in discovery cohort. **Table S3.** Changes in 15 metabolites between non-severe CAP patients and controls in discovery cohort. **Table S4.** Most significant differential metabolites changed between any two groups in discovery cohort. **Table S5.** Changes in eight metabolites between CAP patients and controls in validation cohort. **Table S6.** Correlation analysis between five metabolites for CAP and clinical parameters. (DOCX 43 kb)

## Abbreviations

APACHE II: Acute Physiology and Chronic Health Evaluation II
AUC: Area under the curve
CAP: Community-acquired pneumonia
CRP: C-reactive protein
CURB-65: Confusion, urea level, respiratory rate, blood pressure, and age > 65 years
CV: Coefficient of variation
CV-ANOVA: Cross-validation analysis of variance
DHEA-S: Dehydroepiandrosterone sulfate
ESI: Election spray ionization
ESR: Erythrocyte sedimentation rate
FC: Fold change
FDR: False discovery rates
IQR: Interquartile range
LC-MS: Liquid chromatography–mass spectrometry
OPLS-DA: Orthogonal partial least squares discriminant analysis
PaO_2_/FiO_2_: Ratio of arterial oxygen tension to inspired oxygen fraction
PCA: Principal component analysis
PCT: Procalcitonin
PSI: Pneumonia severity index
ROC: Receiver operating characteristic
VIP: Variable importance in the projection
WBC: White blood cell
