# Supplementary material for: Metabolic profiles in community-acquired pneumonia: developing assessment tools for disease severity
Source: Crit Care. 2018 May 14;22:130. doi: 10.1186/s13054-018-2049-2 (PMC5952829; doi:10.1186/s13054-018-2049-2)
Supplement: Supplementary file 1 — Supplemental methods. Liquid chromatography–mass spectrometry (LC-MS) analysis. (DOCX 19 kb) [file 13054_2018_2049_MOESM1_ESM.docx]

**Additional file 1**

**Supplemental Methods**

**Liquid chromatography-mass spectrometry (LC-MS) analysis**

Samples were analysed using an Ultimate 3000 UHPLC (Dionex) system coupled to a Thermo Q-Exactive (Orbitrap) mass spectrometer (Thermo Fisher Scientific, San Jose, CA, USA).

In positive mode (ESI+), ACQUITY UPLC BEH Amide column (1.7 μm, 2.1×100 mm, Waters) was used. Mobile phase A was prepared by dissolving 0.63 g of ammonium formate in 50 mL of HPLC-grade water, then adding 950 mL of high performance liquid chromatography (HPLC)-grade acetonitrile and 1 μLof formic acid. Mobile phase B was prepared by dissolving 0.63 g of ammonium formate in 500 mL of HPLC-grade water, followed by 500 mL of HPLC-grade acetonitrile and 1 μL formic acid. The linear gradient was set as follows: 0 min, 1% B; 2 min, 1% B; 3.5 min, 20% B; 17 min, 80% B; 17.5 min, 99% B; 19 min, 99% B; 19.1 min, 1% B; 22 min, 1% B. In negative mode (ESI-), BEH Amide column was also applied (1.7 μm, 2.1×100 mm, Waters). Mobile phase A was prepared by dissolving 0.77g of ammonium acetate in 50 mL of HPLC-grade water, then adding 950 mL of HPLC-grade acetonitrile. Adjust pH to 9.0 with ammonium hydroxide solution. Mobile B was prepared by dissolving 0.77 g of ammonium acetate in 500 mL HPLC-grade water, subsequently, adding 500 mL HPLC-grade acetonitrile and adjusting pH to 9.0 with ammonium hydroxide solution. And the linear gradient was as follows: 0 min, 5% B; 2 min 5% B; 4 min, 20% B; 18 min, 85% B; 19 min, 95% B; 21 min, 95% B; 21.1 min, 5% B; 25 min, 5% B.

The detailed mass spectrometer parameters are as follows: spray voltage, 3.5 KV for positive and 2.5 KV for negative mode; capillary temperature, 275°C for positive and 320°C for negative mode; sheath gas flow rate (arb), 35; aux gas flow rate (arb), 8; mass range (m/z), 70-1050 for positive and 80-1200 for negative mode; full MS resolution, 70, 000; MS/MS resolution, 17,500; top N, 10; NCE, 15/30/45; duty cycle, 1.2s.
