# Supplementary material for: Metabolic profiles in community-acquired pneumonia: developing assessment tools for disease severity
Source: Crit Care. 2018 May 14;22:130. doi: 10.1186/s13054-018-2049-2 (PMC5952829; doi:10.1186/s13054-018-2049-2)
Supplement: Supplementary file 2 — Supplemental Figures. Figure S1. Metabolite base peak chromatograms of serum samples from a patient in three groups: a non-severe CAP; b severe CAP; c controls. Figure S2. S-plots identifying putative biomarkers on the basis of OPLS-DA models: a CAP patients versus controls; b severe CAP versus non-severe CAP patients. Figure S3. Box–whisker plots of relative intensity of 15 metabolites changed in CAP patients compared to controls. Horizontal line represents median; bottom and top of the box represent 25th and the 75th percentiles; whiskers represent 5% and 95% percentiles. *FDR < 0.05, **FDR < 0.001. NSCAP non-severe CAP, SCAP severe CAP. (DOCX 7213 kb) [file 13054_2018_2049_MOESM2_ESM.docx]

**Additional file 2**


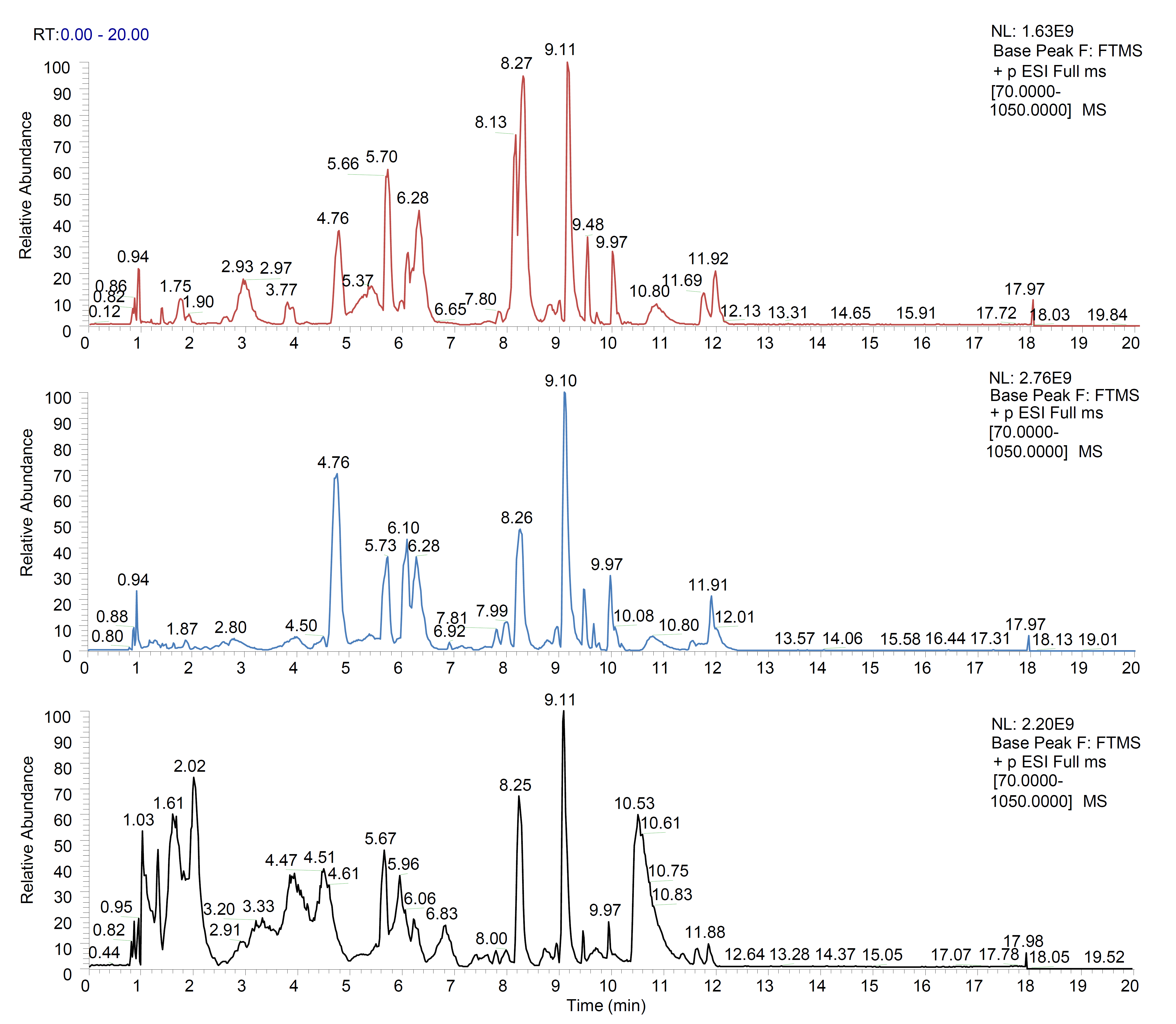


**b**

**c**

**a**

**Fig. S1.** Metabolites base peak chromatogram of serum sample from a patient in three groups. **a** non-severe CAP. **b** severe CAP. **c** controls

**a**


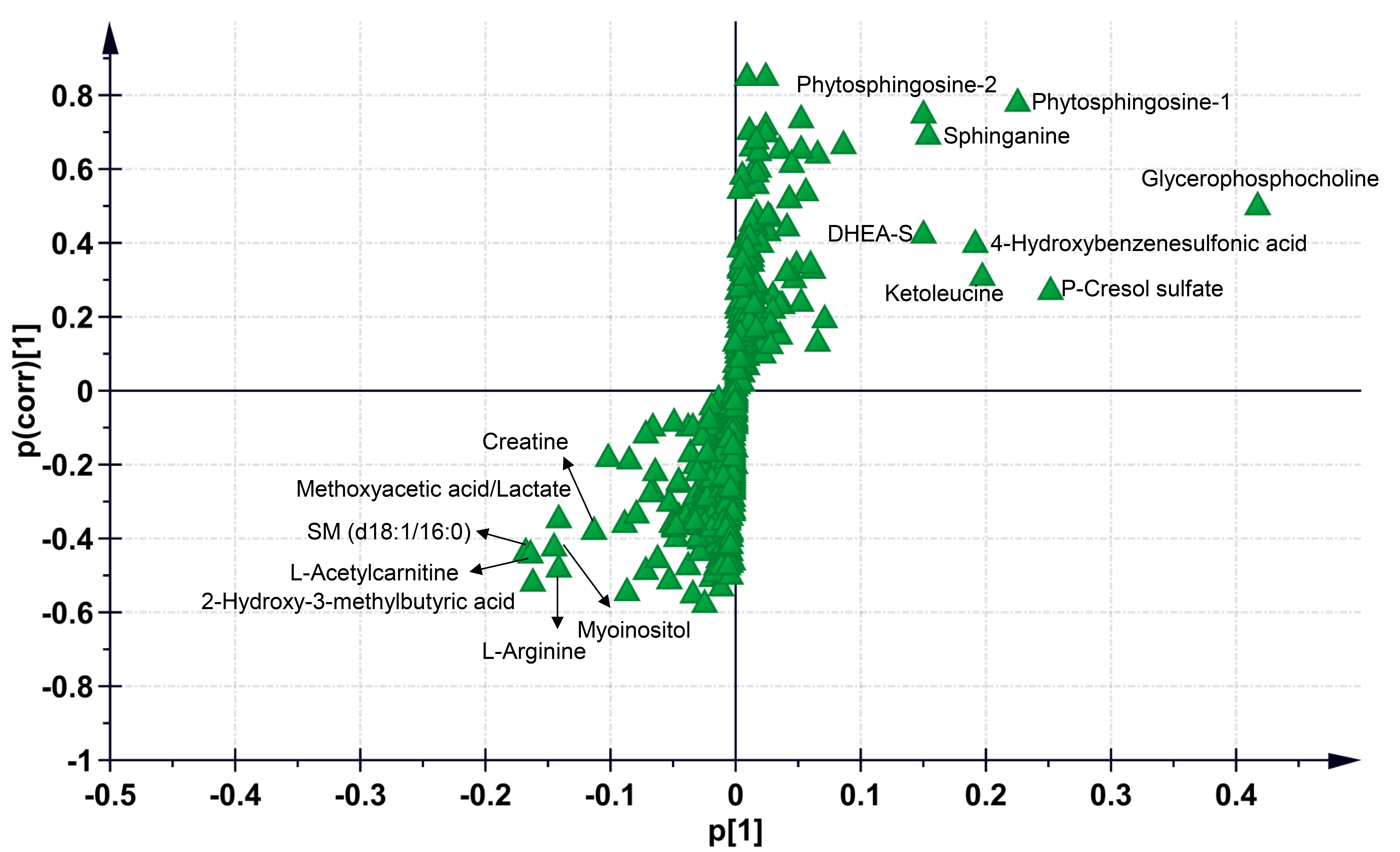


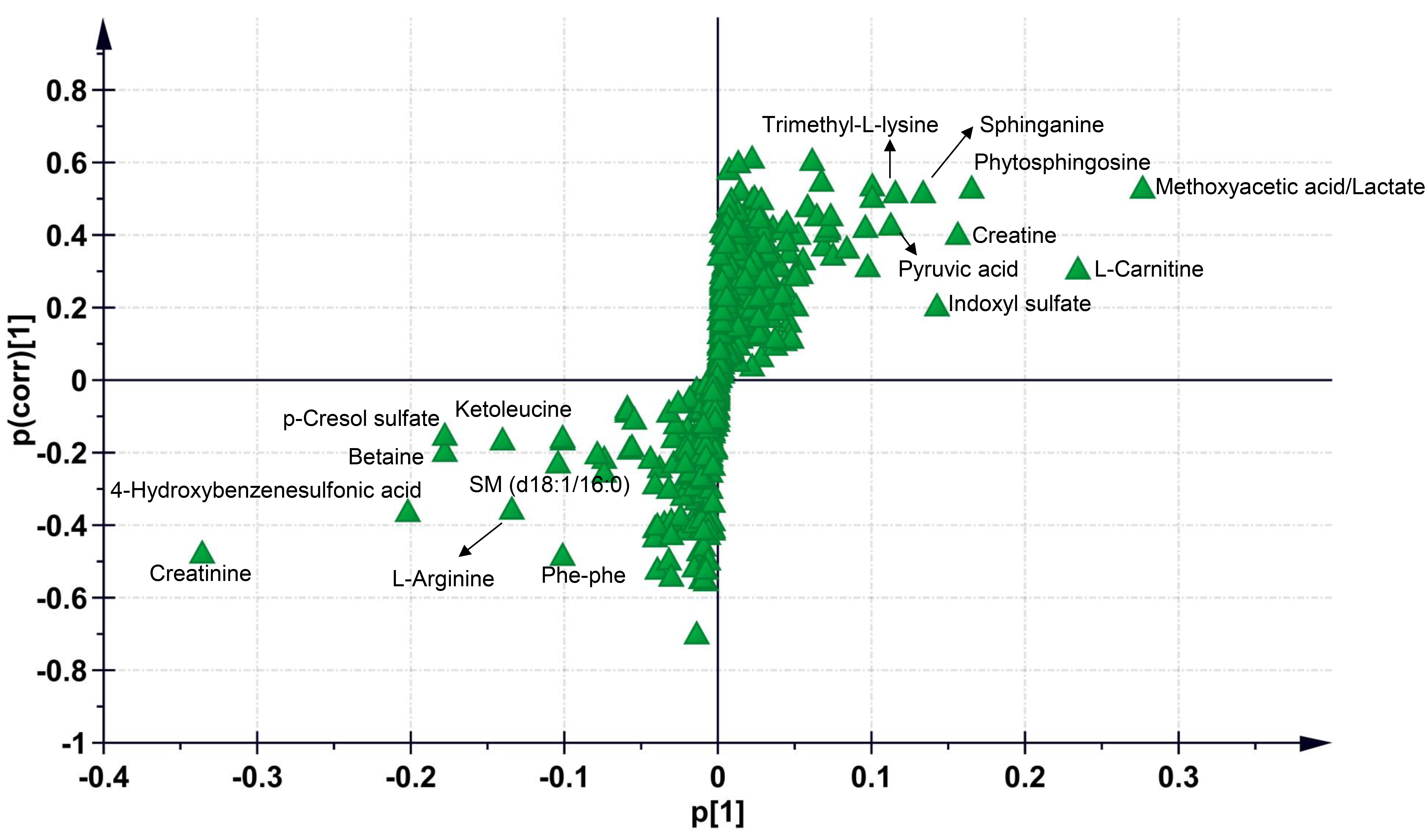


**b**

**Fig. S2.** S-plots identified putative biomarkers on the basis of OPLS-DA models. **a** CAP *vs.* controls. **b** Severe CAP *vs.* non-severe CAP


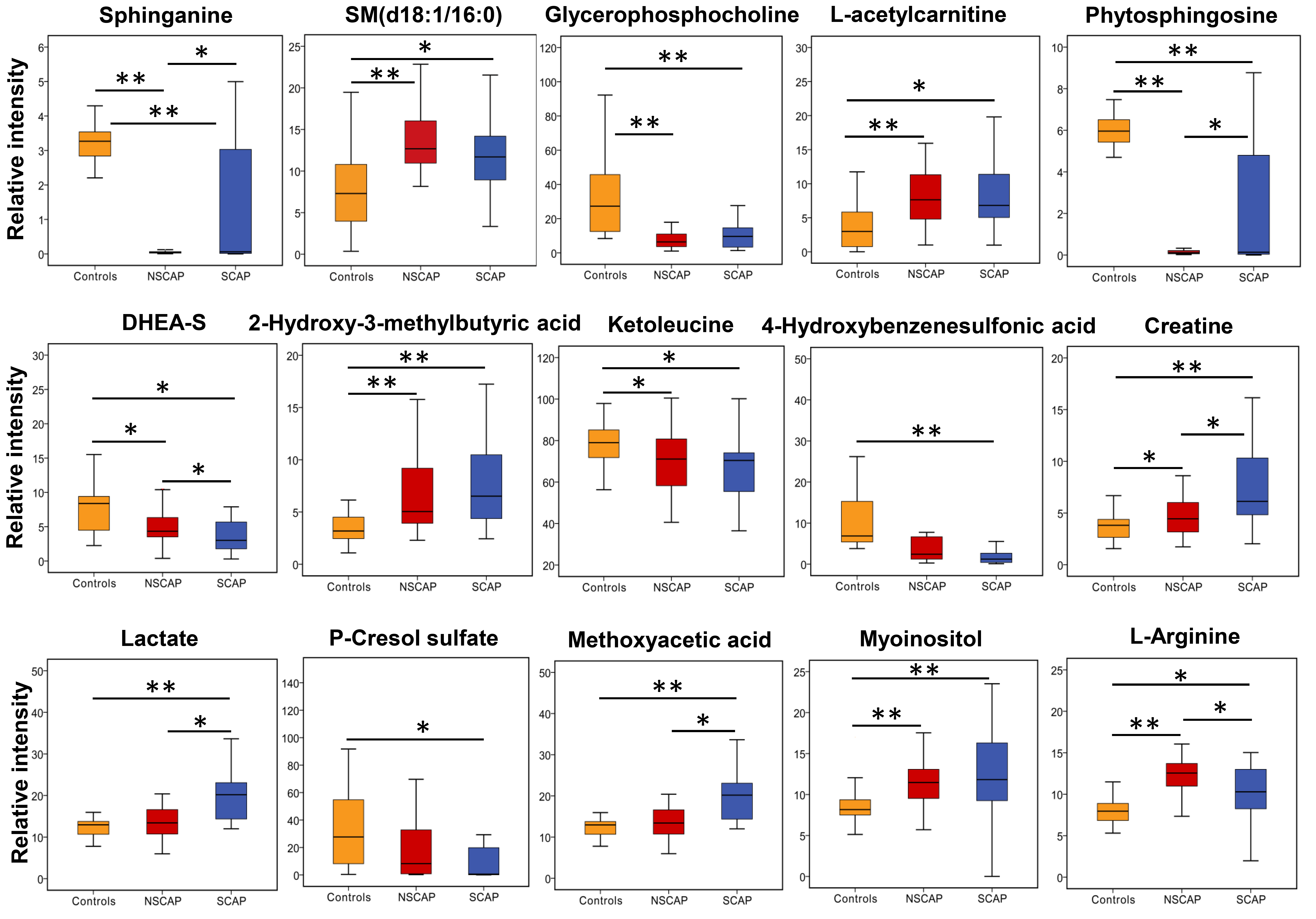


**Fig. S3.** Box-whisker plots of the relative intensity of fifteen metabolites changed in CAP compared to controls. The horizontal line represents the median; the bottom and the top of the box represent the 25th and the 75th percentiles; whiskers represent 5% and 95% percentiles. *NSCAP* non-severe CAP, *SCAP* severe CAP, *DHEA-S* dehydroepiandrosterone sulfate. * FDR < 0.05, ** FDR < 0.001
