# Supplementary material for: Metabolic profiles in community-acquired pneumonia: developing assessment tools for disease severity
Source: Crit Care. 2018 May 14;22:130. doi: 10.1186/s13054-018-2049-2 (PMC5952829; doi:10.1186/s13054-018-2049-2)
Supplement: Supplementary file 3 — Supplemental tables. Table S1. Changes in eight metabolites between severe and non-severe CAP patients in discovery cohort. Table S2. Changes in 17 metabolites between severe CAP patients and controls in discovery cohort. Table S3. Changes in 15 metabolites between non-severe CAP patients and controls in discovery cohort. Table S4. Most significant differential metabolites changed between any two groups in discovery cohort. Table S5. Changes in eight metabolites between CAP patients and controls in validation cohort. Table S6. Correlation analysis between five metabolites for CAP and clinical parameters. (DOCX 43 kb) [file 13054_2018_2049_MOESM3_ESM.docx]

**Additional file 3**

**Table S1.** Changes in 8 metabolites between severe and non-severe CAP patients in the discovery cohort

| Metabolites | VIP | FDR ^a^ | FC ^b^ | Tendency |
| --- | --- | --- | --- | --- |
| Phytosphingosine | 3.6201 | 1.80E-03 | 12.7363 | Controls>SCAP>NSCAP |
| Sphinganine | 2.9288 | 3.26E-03 | 21.9804 | Controls>SCAP>NSCAP |
| Creatine | 3.5786 | 9.60E-03 | 1.6264 | SCAP>NSCAP>Controls |
| L-arginine | 3.3319 | 2.47E-02 | 0.8191 | NSCAP > SCAP >Controls |
| Lactate | 6.5326 | 1.60E-03 | 1.4637 | SCAP>NSCAP>Controls |
| Methoxyacetic acid | 6.5326 | 1.60E-03 | 1.4637 | SCAP>NSCAP>Controls |
| 4-Hydroxybenzenesulfonic acid | 4.5414 | 4.98E-02 | 0.3329 | Controls> NSCAP > SCAP |
| DHEA-S | 2.7664 | 1.18E-02 | 0.5819 | Controls> NSCAP > SCAP |

*CAP* community-acquired pneumonia, *SCAP* severe CAP, *NSCAP* non-severe CAP, *DHEA-S* dehydroepiandrosterone sulfate, *VIP* variable importance in the projection, *FDR* false discovery rate, *FC* fold change

^a^ FDR for comparisons between severe CAP and non-severe CAP group

^b^ FC indicates the ratio of relative high intensity present in severe CAP to non-severe CAP patients

**Table S2.** Changes in 17 metabolites between severe CAP and controls in the discovery cohort

| Metabolites | VIP | FDR | FC^a^ | Change in severe CAP/controls |
| --- | --- | --- | --- | --- |
| 4-Hydroxybenzenesulfonic acid | 5.3378 | 2.61E-05 | 0.1879 | Down |
| Glycerophosphocholine | 9.5040 | 7.51E-04 | 0.3111 | Down |
| Phytosphingosine | 3.4091 | 5.24E-07 | 0.4031 | Down |
| p-Cresol sulfate | 8.7028 | 1.83E-02 | 0.4140 | Down |
| Sphinganine | 2.1336 | 5.11E-04 | 0.4747 | Down |
| DHEA-S | 3.2276 | 8.18E-03 | 0.5587 | Down |
| Ketoleucine | 6.4351 | 2.97E-03 | 0.8439 | Down |
| L-Arginine | 2.3334 | 6.38E-03 | 1.2477 | Up |
| Palmitoyl sphingomyelin (SM(d18:1/16:0)) | 3.0691 | 1.43E-02 | 1.4724 | Up |
| D-(+)-Glucose | 3.5103 | 7.93E-04 | 1.4725 | Up |
| Myoinositol | 3.5103 | 7.93E-04 | 1.4725 | Up |
| L-Phenylalanine | 2.5692 | 2.25E-05 | 1.4922 | Up |
| Lactate | 5.1844 | 4.13E-06 | 1.6029 | Up |
| Methoxyacetic acid | 5.1844 | 4.13E-06 | 1.6029 | Up |
| Creatine | 3.4995 | 6.93E-05 | 2.0577 | Up |
| L-Acetylcarnitine | 3.7363 | 1.39E-03 | 2.1657 | Up |
| 2-Hydroxy-3-methylbutyric acid | 3.5203 | 7.12E-06 | 2.1660 | Up |

*VIP* variable importance in the projection, *FDR* false discovery rate, *FC* fold change

a FC indicates the ratio of relative high intensity present in severe CAP patients to controls

**Table S3.** Changes in 15 metabolites between non-severe CAP and controls in the discovery cohort

| Metabolites | VIP | FDR | FC^a^ | Change in non-severe CAP/controls |
| --- | --- | --- | --- | --- |
| Sphinganine | 4.3259 | 1.42E-31 | 0.0216 | Down |
| Phytosphingosine | 5.8814 | 6.86E-45 | 0.0316 | Down |
| Glycerophosphocholine | 10.7827 | 2.13E-04 | 0.2869 | Down |
| DHEA-S | 3.1673 | 3.08E-03 | 0.5652 | Down |
| Ketoleucine | 5.8843 | 4.69E-02 | 0.9060 | Down |
| Betaine | 5.5082 | 4.44E-02 | 1.1326 | Up |
| Creatinine | 5.1006 | 1.08E-02 | 1.1504 | Up |
| D-(+)-Glucose | 3.7400 | 2.52E-04 | 1.3649 | Up |
| Myoinositol | 3.7400 | 2.52E-04 | 1.3649 | Up |
| Hypoxanthine | 3.3874 | 4.04E-02 | 1.4614 | Up |
| L-Arginine | 4.3015 | 3.64E-09 | 1.5232 | Up |
| Palmitoyl sphingomyelin (SM(d18:1/16:0)) | 4.2106 | 1.18E-05 | 1.7734 | Up |
| 2-Hydroxy-3-methylbutyric acid | 3.2443 | 6.72E-05 | 1.9305 | Up |
| L-Acetylcarnitine | 3.9509 | 9.47E-05 | 2.1009 | Up |
| Valproic acid | 2.6290 | 1.06E-02 | 2.3206 | Up |

*VIP* variable importance in the projection, *FDR* false discovery rate, *FC* fold change

a FC indicates the ratio of relative high intensity present in non-severe CAP patients to controls

**Table S4.** Most significant differential metabolites changed between any two groups in the discovery cohort

| SCAP *vs.* NSCAP | |  | SCAP *vs.* controls | |  | NSCAP *vs.* controls | |
| --- | --- | --- | --- | --- | --- | --- | --- |
| Decreased in SCAP | Increased in SCAP |  | Decreased in SCAP | Increased in SCAP |  | Decreased in NSCAP | Increased in NSCAP |
| DHEA-S | Creatine |  | DHEA-S | L-Arginine |  | Sphinganine | Betaine |
| L-Arginine | Lactate |  | Glycerophosphocholine | SM (d18:1/16:0) |  | Phytosphingosine | Creatinine |
| 4-Hydroxybenzenesulfonic acid | Methoxyacetic acid |  | Phytosphingosine | D-(+)-Glucose |  | Glycerophosphocholine | D-(+)-Glucose |
|  | Phytosphingosine |  | P-Cresol sulfate | Myoinositol |  | DHEA-S | Myoinositol |
|  | Sphinganine |  | Sphinganine | L-phenylalanine |  | Ketoleucine | Hypoxanthine |
|  |  |  | Ketoleucine | Lactate |  |  | L-Arginine |
|  |  |  | 4-Hydroxybenzenesulfonic acid | Methoxyacetic acid |  |  | SM (d18:1/16:0) |
|  |  |  |  | Creatine |  |  | L-acetylcarnitine |
|  |  |  |  | L-Acetylcarnitine |  |  | Valproic acid |
|  |  |  |  | 2-Hydroxy-3-methylbutyric acid |  |  | 2-Hydroxy-3-methylbutyric acid |

*SCAP* severe CAP, *NSCAP* non-severe CAP, *DHEA-S* dehydroepiandrosterone sulfate

**Table S5.** Changes in 8 metabolites between CAP and controls in the validation cohort

| Metabolites | Concentrations (μg/mL) | | *p* value | Changes in CAP/controls |
| --- | --- | --- | --- | --- |
|  | CAP (n = 51) | Controls (n = 22) |  |  |
| Sphinganine | 0.025 (0.019–0.040) | 0.049 (0.045–0.055) | <0.001 | Down |
| Creatine | 3.447 (2.143–5.672) | 3.463 (2.585–4.064) | 0.838 | Down |
| L-arginine | 5.318 (4.469–6.704) | 4.714 (3.833–5.644) | 0.049 | Up |
| Lactate | 120.020  (90.564–153.052) | 96.719  (73.753–131.420) | 0.047 | Up |
| L-acetylcarnitine | 0.068 (0.037–0.136) | 0.086 (0.053–0.116) | 0.682 | Down |
| Glycerophosphocholine | 4.789 (2.834–8.055) | 5.841 (3.979–11.276) | 0.861 | Down |
| DHEA-S | 0.438 (0.342–0.728) | 0.898 (0.692–1.170) | <0.001 | Down |
| P-Cresol sulfate | 0.085 (0.008–0.431) | 1.093 (0.221–1.511) | <0.001 | Down |

*CAP* community-acquired pneumonia, *DHEA-S* dehydroepiandrosterone sulfate, *SCAP* severe CAP, *NSCAP* non-severe CAP

**Table S6.** Correlation analysis between five metabolites for CAP and clinical parameters

| Variables | | Sphinganine | L-Arginine | DHEA-S | Lactate | P-Cresol sulfate | WBC | NE% | ESR | CRP | PCT | PSI | CURB-65 | APACHE II |
| --- | --- | --- | --- | --- | --- | --- | --- | --- | --- | --- | --- | --- | --- | --- |
| Sphinganine | r* | 1 | -0.100 | -0.346 | 0.318 | 0.015 | 0.181 | 0.336 | 0.519 | 0.340 | 0.200 | 0.570 | 0.456 | 0.442 |
|  | *p* value | —— | 0.487 | 0.013 | 0.023 | 0.917 | 0.203 | 0.016 | 0.000 | 0.015 | 0.159 | 0.000 | 0.001 | 0.001 |
| L-Arginine | r | -0.100 | 1 | 0.027 | -0.206 | 0.156 | 0.033 | -0.283 | -0.181 | -0.116 | -0.188 | -0.110 | -0.119 | -0.177 |
|  | *p* value | 0.487 | —— | 0.851 | 0.146 | 0.273 | 0.817 | 0.044 | 0.203 | 0.417 | 0.187 | 0.442 | 0.406 | 0.213 |
| DHEA-S | r | -0.346 | 0.027 | 1 | -0.144 | -0.251 | -0.246 | -0.255 | -0.377 | -0.272 | -0.243 | -0.379 | -0.364 | -0.397 |
|  | *p* value | 0.013 | 0.851 | —— | 0.312 | 0.075 | 0.081 | 0.071 | 0.006 | 0.054 | 0.086 | 0.006 | 0.009 | 0.004 |
| Lactate | r | 0.318 | -0.206 | -0.144 | 1 | 0.082 | 0.213 | 0.299 | 0.300 | 0.251 | 0.298 | 0.491 | 0.408 | 0.430 |
|  | *p* value | 0.023 | 0.146 | 0.312 | —— | 0.567 | 0.134 | 0.033 | 0.033 | 0.076 | 0.034 | 0.000 | 0.003 | 0.002 |
| P-Cresol sulfate | r | 0.015 | 0.156 | -0.251 | 0.082 | 1 | 0.097 | 0.132 | 0.112 | 0.105 | 0.098 | 0.298 | 0.296 | 0.393 |
|  | *p* value | 0.917 | 0.273 | 0.075 | 0.567 | —— | 0.500 | 0.357 | 0.434 | 0.465 | 0.495 | 0.034 | 0.035 | 0.004 |
| WBC | r | 0.181 | 0.033 | -0.246 | 0.213 | 0.097 | 1 | 0.474 | 0.416 | 0.360 | 0.157 | 0.336 | 0.280 | 0.404 |
|  | *p* value | 0.203 | 0.817 | 0.081 | 0.134 | 0.500 | —— | 0.000 | 0.002 | 0.010 | 0.273 | 0.016 | 0.046 | 0.003 |
| NE% | r | 0.336 | -0.283 | -0.255 | 0.299 | 0.132 | 0.474 | 1 | 0.261 | 0.636 | 0.459 | 0.543 | 0.428 | 0.582 |
|  | *p* value | 0.016 | 0.044 | 0.071 | 0.033 | 0.357 | 0.000 | —— | 0.064 | 0.000 | 0.001 | 0.000 | 0.002 | 0.000 |
| ESR | r | 0.519 | -0.181 | -0.377 | 0.300 | 0.112 | 0.416 | 0.261 | 1 | 0.377 | 0.132 | 0.427 | 0.347 | 0.381 |
|  | *p* value | 0.000 | 0.203 | 0.006 | 0.033 | 0.434 | 0.002 | 0.064 | —— | 0.006 | 0.356 | 0.002 | 0.013 | 0.006 |
| CRP | r | 0.340 | -0.116 | -0.272 | 0.251 | 0.105 | 0.360 | 0.636 | 0.377 | 1 | 0.493 | 0.445 | 0.342 | 0.503 |
|  | *p* value | 0.015 | 0.417 | 0.054 | 0.076 | 0.465 | 0.010 | 0.000 | 0.006 | —— | 0.000 | 0.001 | 0.014 | 0.000 |
| PCT | r | 0.200 | -0.188 | -0.243 | 0.298 | 0.098 | 0.157 | 0.459 | 0.132 | 0.493 | 1 | 0.272 | 0.252 | 0.450 |
|  | *p* value | 0.159 | 0.187 | 0.086 | 0.034 | 0.495 | 0.273 | 0.001 | 0.356 | 0.000 | —— | 0.053 | 0.075 | 0.001 |
| PSI | r | 0.570 | -0.110 | -0.379 | 0.491 | 0.298 | 0.336 | 0.543 | 0.427 | 0.445 | 0.272 | 1 | 0.874 | 0.779 |
|  | *p* value | 0.000 | 0.442 | 0.006 | 0.000 | 0.034 | 0.016 | 0.000 | 0.002 | 0.001 | 0.053 | —— | 0.000 | 0.000 |
| CURB-65 | r | 0.456 | -0.119 | -0.364 | 0.408 | 0.296 | 0.280 | 0.428 | 0.347 | 0.342 | 0.252 | 0.874 | 1 | 0.824 |
|  | *p* value | 0.001 | 0.406 | 0.009 | 0.003 | 0.035 | 0.046 | 0.002 | 0.013 | 0.014 | 0.075 | 0.000 | —— | 0.000 |
| APACHE II | r | 0.442 | -0.177 | -0.397 | 0.430 | 0.393 | 0.404 | 0.582 | 0.381 | 0.503 | 0.450 | 0.779 | 0.824 | 1 |
|  | *p* value | 0.001 | 0.213 | 0.004 | 0.002 | 0.004 | 0.003 | 0.000 | 0.006 | 0.000 | 0.001 | 0.000 | 0.000 | —— |

*CAP* community-acquired pneumonia, *DHEA-S* dehydroepiandrosterone sulfate, *WBC* white blood cell, *NE%* percentage of neutrophils, *ESR* erythrocyte sedimentation rate, *CRP* C reactive protein, *PCT* procalcitonin, *CURB-65* confusion, urea level, respiratory rate, blood pressure, and age≥65years, *PSI* pneumonia severity index, *APACHE II* Acute Physiology and Chronic Health Evaluation II

* r: Pearson’s correlation coefficient
